# Novo Modelo Incremental para Predição de Mortalidade na Hipertensão Pulmonar Pré-Capilar

**DOI:** 10.36660/abc.20230669

**Published:** 2024-07-23

**Authors:** Andressa Alves de Carvalho, Wanessa Alves de Carvalho, Eliauria Rosa Martins, Agostinho Hermes de Medeiros, Fernando Bacal, Marcelo Dantas Tavares de Melo

**Affiliations:** 1 Universidade Federal da Paraíba João Pessoa PB Brasil Universidade Federal da Paraíba, João Pessoa, PB – Brasil; 2 Universidade de São Paulo Faculdade de Medicina Hospital das Clínicas Instituto do Coração São Paulo SP Brasil Universidade de São Paulo Faculdade de Medicina Hospital das Clínicas Instituto do Coração, São Paulo, SP – Brasil

**Keywords:** Hipertensão Pulmonar, Eritrócitos, Deformação Longitudinal Global

## Abstract

**Fundamento:**

Na hipertensão pulmonar (HP), é necessária a identificação de marcadores prognósticos de fácil obtenção associados com disfunção do ventrículo direito (VD) e sobrevida.

**Objetivo:**

Avaliar a associação do índice de anisocitose eritrocitária (RDW, do inglês red cell distribution width) com parâmetros ecocardiográficos e sobrevida em pacientes com HP pré-capilar, com o desenvolvimento de um modelo de predição de mortalidade.

**Métodos:**

Estudo observacional, longitudinal, prospectivo, conduzido entre maio de 2019 e dezembro de 2022. Trinta e quatro pacientes com HP pré-capilar submeteram-se à realização de ecocardiograma bidimensional e hemograma. Um ponto de corte de 14,5% foi adotado para definir o RDW como alterado (≥14,5%) ou normal (<14,5%). Valores de p<0,05 foram considerados significativos.

**Resultados:**

O RDW médio foi 14,4%. Houve uma diferença significativa na saturação periférica de oxigênio (SpO_2_) (p=0,028), *strain* do VD (p=0,047) e derrame pericárdico (p=0,002) entre os grupos com RDW normal e elevado. Durante um período mediano de 15 meses, 20,6% dos pacientes foram a óbito. Os pacientes com RDW aumentado tiveram uma sobrevida global mais curta (44,7%, log-rank p=0,019), sendo um preditor de mortalidade na regressão univariada de Cox. A adição do *strain* do VD < 16% e da SpO_2_ ≤93% ao modelo incluindo somente RDW mostrou valor incremental na predição de mortalidade (χ^2^=8,2, p=0,049; χ^2^=12,4, p=0,041), com área sob a curva ROC (do inglês, Receiver Operating Characteristic) aumentada (0,729 vs. 0,837 vs. 0,909) e probabilidade de sobrevida diminuída (44.7% vs. 35.6% vs. 25%, log-rank p=0,019).

**Conclusões:**

O RDW fornece informações sobre a gravidade da HP pré-capilar pela sua correlação com parâmetros ecocardiográficos de disfunção do VD e mortalidade, a qual é melhor predita por um modelo incluindo RDW, *strain* do VD e SpO_2_.

## Graphical abstract

**Figure:**
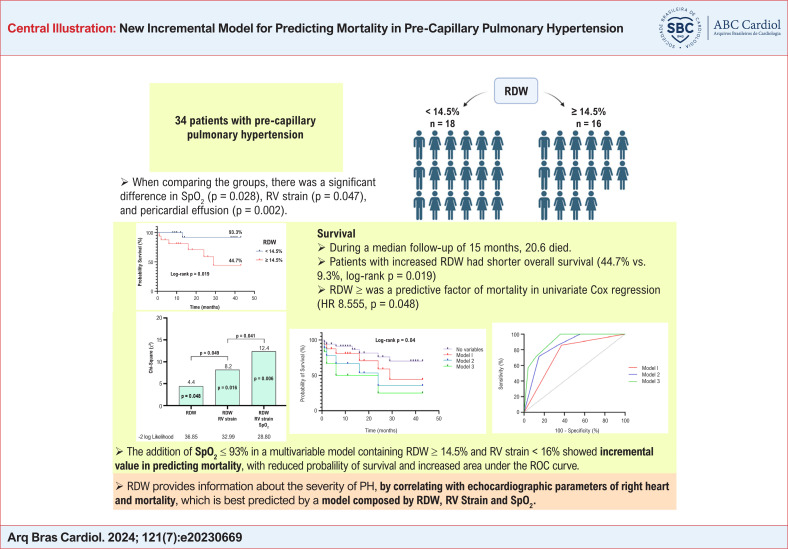
RDW: Red Cell Distribution Width; VD: ventrículo direito; SpO_2_: saturação de oxigênio arterial periférico.

## Introdução

A hipertensão pulmonar (HP) é definida pela presença de uma pressão arterial pulmonar média igual ou maior a 20 mmHg, avaliada por cateterismo cardíaco.^1^ A HP pré-capilar é caracterizada por uma pressão de oclusão da artéria pulmonar igual ou menor a 15 mmHg e inclui pacientes dos grupos 1, 3 e 4, alguns pacientes do grupo 5 e, raramente, pacientes do grupo 2 que apresentam uma combinação de HP pré-capilar e pós-capilar.^2^

Em estágios avançados, a HP pode levar à hipertrofia ventricular direita e à insuficiência cardíaca terminal. Nesse sentido, existe uma clara necessidade de se identificar marcadores prognósticos de fácil obtenção associados com disfunção ventricular direita e sobrevida em pacientes com HP.

O índice de anisocitose eritrocitária (RDW, do inglês *red cell distribution width*) é um dos parâmetros obtidos do hemograma e mede a variabilidade no volume das hemácias circulantes. Quando elevada, reflete a presença de uma disfunção na eritropoiese, maior destruição, ou uma meia vida reduzida das hemácias.^3^ A causa mais comum de elevação do RDW é anemia,^4^ mas estudos recentes mostram que seu aumento está associado com várias condições, tais como HP, na qual tem um valor prognóstico.^5^

Não existem mecanismos claros para explicar a relação entre RDW e doença cardiovascular. Uma das principais hipóteses é o papel da inflamação crônica, que causa mielossupressão, reduz a síntese renal da eritropoietina e estimula a apoptose de precursores eritroides na medula óssea, aumentando a anisocitose.^6^

O objetivo deste estudo foi avaliar a associação do RDW com parâmetros clínicos, laboratoriais e ecocardiográficos em pacientes com HP pré-capilar, bem como seu valor prognóstico na sobrevida, com o desenvolvimento de um modelo incremental para a predição de mortalidade.

## Métodos

### Delineamento e população do estudo

Este é um estudo observacional, longitudinal e prospectivo, conduzido de maio de 2019 a dezembro de 2022. Foram incluídos pacientes com um diagnóstico de HP pré-capilar confirmado por cateterismo cardíaco direito, com idade maior que 18 anos, e acompanhados no ambulatório de pneumologia de um hospital universitário.

Os critérios de exclusão foram: a) pacientes com HP pós-capilar; b) presença de congestão hemodinâmica no ecocardiograma (E/E’>14), disfunção diastólica grau 2 ou 3, ou fração de ejeção ventricular esquerda reduzida; c) doença cardíaca estrutural ou valvular do lado esquerdo; d) cardiopatia congênita corrigida ou não; e) janela ecocardiográfica inadequada; f) mulheres grávidas; g) recusa em assinar o termo de consentimento.

O estudo foi aprovado pelo comitê de ética do Centro de Ciências Médicas da Universidade Federal da Paraíba (número 3,616,337, CAAE: 21291419.6.0000.8069).

### Ecocardiografia

O exame de ecocardiografia foi realizado usando um aparelho GE Vivid T8 com transdutor de 2,5 MHz M4h-5. As imagens foram adquiridas com o paciente em decúbito lateral esquerdo, segundo recomendações da *American Society of Echocardiography*.^7^ Foram adquiridas imagens de vídeo correspondentes a três ciclos cardíacos. A fração de ejeção foi estimada usando o método biplanar de Simpson.

O *strain* miocárdico foi avaliado em uma *workstation* pelo programa EchoPach V204, e o traçado do endocárdio foi realizado manualmente no final da diástole. A medida foi feita após o examinador verificar a qualidade do traçado da borda endocárdica. Se dois segmentos fossem considerados inadequados, o exame foi excluído do estudo. Na janela apical, foram adquiridas seções focando o ventrículo direito (VD) e o átrio direito (AD), com quantificação média do *strain* da parede livre.

### Exames laboratoriais

O valor de RDW foi obtido do hemograma, realizado até um mês após o ecocardiograma transtorácico bidimensional com Doppler. O RDW foi considerado alterado ou normal de acordo com o ponto de corte (14,5%) adotado na instituição em que o estudo foi conduzido. Ainda, níveis de peptídeo natriurético cerebral (BNP) foram medidos e comparados com o RDW.

### Desfecho

Os participantes foram incluídos em diferentes momentos e acompanhados desde a data do ecocardiograma até o final do período do estudo ou data de óbito. O desfecho foi definido como mortalidade durante o período de seguimento.

### Viés

Para diminuir o risco de viés, todos os exames ecocardiográficos foram realizados por um único examinador, cego quanto à classificação de HP (grupo), e exames laboratoriais foram realizados no laboratório do mesmo centro. As variáveis clínicas foram obtidas durante consulta de rotina pelo mesmo médico atendente.

### Tamanho amostral

O tamanho amostral foi definido por conveniência, incluindo todos os pacientes acompanhados no ambulatório de HP elegíveis para o estudo.

### Análise estatística

As variáveis contínuas foram apresentadas como média e desvio padrão (distribuição normal) ou medianas e intervalos interquartis (distribuição não normal). As variáveis categóricas foram expressas como frequências absolutas e relativas. A normalidade da distribuição dos dados foi avaliada pelo teste de Kolmogorov-Smirnov test.

As variáveis contínuas paramétricas e não paramétricas foram comparadas usando o teste t de Student para amostras independentes e o teste de Mann-Whitney, respectivamente. Comparações de três ou mais grupos de variáveis não paramétricas foram feitas usando o teste de Kruskal-Wallis, com teste post hoc de Dunn. O grau de correlação entre duas variáveis foi determinado pelo coeficiente de correlação de Spearman, dada à ausência de normalidade na distribuição da amostra.

O teste exato de Fisher foi usado para avaliar a associação entre os grupos de RDW (normal/alterado) e variáveis categóricas como mortalidade. A sobrevida livre de eventos foi avaliada usando o método de Kaplan-Meier, e as curvas foram comparadas usando o teste de log-rank.

O método de regressão de Cox foi usado para identificar a associação entre as variáveis e mortalidade, com cálculo do *Hazard Ratio* (HR) e intervalo de confiança (IC) de 95%. As variáveis com p<0,05 na análise univariada foram incluídas no modelo multivariado.

Modelos de Cox seriados determinaram o valor incremental do *strain* do VD e da saturação periférica de oxigênio (SpO_2_) em predizer mortalidade ao adicionar, gradualmente, variáveis ao modelo contendo somente RDW. Um incremento no valor preditivo foi definido como um aumento estatisticamente significativo no teste do qui-quadrado (χ^2^), usando o teste omnibus. A probabilidade log −2 (-2LL) foi calculada para comparar a capacidade das variáveis em predizer o desfecho. A melhora do modelo em cada estágio foi descrita pela diminuição na −2LL.

Áreas sob a curva característica de operação do receptor (ROC) foram desenvolvidas para comparar os modelos. Um p<0,05 foi considerado estatisticamente significativo. Análises estatísticas foram realizadas usando o programa *Statistical Package for the Social Sciences* (SPSS), versão 23. As curvas ROC foram avaliadas com o programa MedCalc, e as figuras geradas pelo programa GraphPad Prism 9.

## Resultados

### Características dos pacientes

Foram incluídos 34 pacientes com HP pré-capilar (Tabela 1). Durante o período do estudo, não houve perda de seguimento. A idade mediana dos participantes foi 49 anos, e 82,4% dos pacientes eram do sexo feminino. De acordo com a classe funcional da Organização Mundial da Saúde, a maioria dos pacientes apresentava grau III.

**Tabela 1:** Parâmetros clínicos, laboratoriais, e ecocardiográficos da população do estudo e grupos com Índice de Anisocitose Eritrocitária (RDW) normal e aumentado

Parâmetros		Todos os pacientes (n = 34)	Pacientes com RDW <14,5% (n = 18)	Pacientes com RDW ≥14,5% (n = 16)	Valor p
Idade (anos)^a^		49 [37,8-66,5]	54 [43,5-68,3]	41 [37-66,5]	0,19*
Mulheres, n (%)		28(82,4)	15(83,3)	13(81,3)	1,0†
IMC (kg/m²)^b^		26,8 ± 5,9	27,7 ± 7,2	25,7 ± 4,0	0,31††
Saturação periférica de oxigênio (%)^a^		94 [92-96]	95,5 [93-97]	92,5 [90-95]	0,028*
**Classe funcional, n (%)**					
	I	2 (5,9)	0	2 (12,5)	0,046†
	II	8 (23,5)	7 (38,9)	1 (6,3)	
	III	24 (70,6)	11 (61,1)	13 (81,3)	
	IV	0	0	0	
**Número de medicamentos em uso, n (%)**					
	0	10(29,4)	5(27,8)	5(31,3)	0,6†
	1	10(29,4)	7(38,9)	3(18,8)	
	2	11(32,4)	5(17,8)	6(37,5)	
	3	3(8,8)	1(5,6)	2(12,5)	
**Etiologia, n (%)**					
	Grupo I	22(64,7)	12(66,7)	10(62,5)	0,88†
	Grupo III	5(14,7)	3(16,7)	2(12,5)	
	Grupo IV	7(20,6)	3(16,7)	4(25)	
Doença do tecido conjuntivo, n (%)		5(14,7)	4(22,2)	1(6,3)	0,32†
Hipertensão arterial pulmonar idiopática, n (%)		15(44,1)	6(33,3)	9(56,3)	0,41†
Hemoglobina (g/dL)^b^		13,8 ± 2,0	13,5 ± 1,3	14,3 ± 2,5	0,51††
Hematócrito (%)^a^		42,5 ± 6,3	41,7 [39,3-43,8]	43,5 [38,5-48,1]	0,4*
BNP (pg/ml)^a^		36,0 [13,2-349]	17,6 [11-109]	161,8 [21-795]	0,09*
TAPSE (m^m^)^b^		17,6 ± 5,7	19,1 ± 6,2	15,9 ± 4,7	0,11††
VRT (m/s)^b^		4,0 ± 1,0	3,9 ± 0,6	4,2 ± 1,3	0,33††
PASP (mmHg)^b^		69,4 ± 23,4	67,2 ± 23,6	71,8 ± 23,7	0,57††
FAC (%)^b^		33,1 ± 13,5	32,9 ± 14,9	33,3 ± 12,3	0,93††
S′ (cm/s)^a^		11,6 ± 3,9	11,0[10-14]	11,0[10-13]	0,42*
TAPSE/PASP (mm/mmHg)^a^		0,29 ± 0,16	0,3 [0,22-0,37]	0,19 [0,17-0,36]	0,06*
Área do AD (cm^2^)^a^		21,1 ± 8,1	18,3 [14,7-26,4]	19,0 [10,3-29,6]	0,81*
Pressão do AD (mmHg)^a^		9,1 ± 9,7	5,0[3-11,5]	8,0[3-15]	0,41*
*Strain* do AD (%)^b^		26,6 ± 17,0	27,6 ± 14,9	25,2 ± 19,8	0,72††
Diâmetro do VD (mm)^b^		41,5 ± 11,0	41,6 ± 7,2	41,5 ± 14,3	0,07††
*Strain* do VD (%)^b^		17,8 ± 6,9	20,0 ± 6,3	15,3 ± 6,8	0,047††
Derrame pericárdico, n (%)		7(20,6)	0	7(43,8)	0,002†
FEVE (%)		70,9 ± 6,6	68,8 ± 6,9	67,4 ± 5,3	0,54††
Massa ventricular esquerda (g)		118,5 ± 37,1	131,1 ± 38,5	100,3 ± 27,4	0,02††
E/e′		6,6 ± 2,2	6,7 ± 1,7	6,3 ± 2,9	0,66††

RDW: Índice de Anisocitose Eritrocitária; IMC: Índice de Massa Corporal; BNP: Peptídeo Natriurético Cerebral; TAPSE: excursão sistólica do plano do anel tricúspide; VRT: Velocidade de Regurgitação Tricúspide; PASP: Pressão Sistólica da Artéria Pulmonar; FAC: Fração de variação da área; S′: pico da velocidade sistólica do anel tricúspide; AD: Átrio Direito; VD: Ventrículo Direito; FEEVE: Fração de Ejeção do Ventrículo Esquerdo; E/e′: razão entre a velocidade diastólica E do fluxo mitral e a velocidade diastólica e’ do anel mitral. Valores expressos em mediana e intervalo interquartil; Valores expressos em média e desvio padrão;

* Teste de Mann-Whitney;

† Teste exato de Fisher;

†† Teste t de Student.

Em relação à terapia, 61,8% dos pacientes tomavam um ou dois medicamentos. Pacientes sem tratamento adequado haviam sido recentemente encaminhados para tratamento. A maioria estava usando um inibidor da fosfodiesterase-5, seguido de um antagonista de receptor de endotelina e/ou um análogo de prostaciclina (Tabela S1).

As etiologias mais comuns da HP foram hipertensão arterial pulmonar (HAP) idiopática, tromboembolismo pulmonar, doença do tecido conjuntivo e doença pulmonar obstrutiva crônica (DPOC) (Tabela S1).

### Resultados laboratoriais e ecocardiográficos

Na avaliação laboratorial e ecocardiográfica (Tabela 1), o valor mediano do RDW foi próximo ao limite superior de normalidade. Nenhum dos pacientes apresentou disfunção cardíaca esquerda, e a minoria apresentou derrame pericárdico.

**Figura 1:**
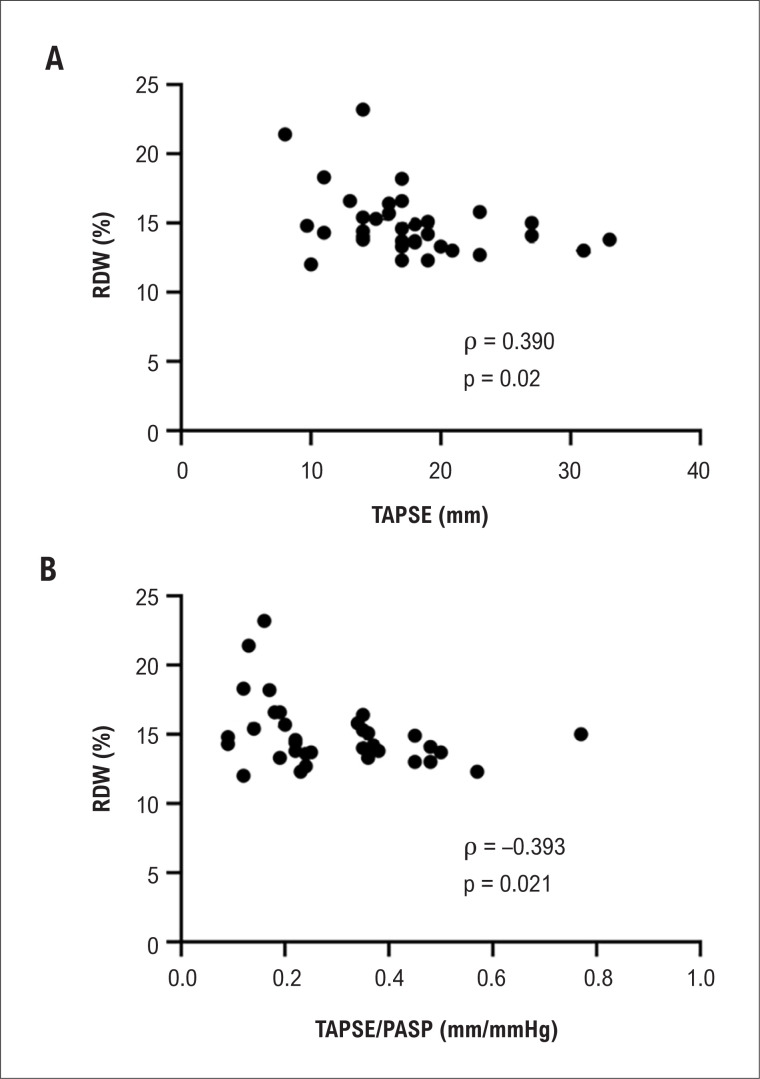
Correlação do Índice de Anisocitose Eritrocitária (RDW) com excursão sistólica do plano do anel tricúspide (TAPSE) (1A) e com TAPSE/ Pressão Sistólica da Artéria Pulmonar (PSAP) (TAPSE/PSAP) (1B).

O RDW correlacionou-se inversamente com excursão sistólica do plano do anel tricúspide (TAPSE) e com TAPSE/ Pressão Sistólica da Artéria Pulmonar (PSAP) (Figura 1), mas não se correlacionou com nenhum outro parâmetro ecocardiográfico (Tabela 2). O RDW não mostrou correlação com BNP nem com a hemoglobina.

**Tabela 2:** Correlação de Spearman entre Índice de Anisocitose Eritrocitária (RDW) e parâmetros ecocardiográficos e laboratoriais

Variáveis ecocardiográficas e laboratoriais	RDW	
	ρ (correlation coefficient)	Valor p
Hemoglobina	0,218	0,23
Hematócrito	0,201	0,27
BNP	0,295	0,15
TAPSE	-0,390	0,02
TRV	0,162	0,36
PASP	0,160	0,37
FAC	-0,032	0,86
S′	-0,091	0,61
TAPSE/PASP	-0,393	0,021
Área do AD	-0,054	0,78
Pressão do AD	0,167	0,35
*Strain* do AD	-0,025	0,89
Diâmetro do VD	0,001	0,99
*Strain* do VD	-0,290	0,09

RDW: índice de anisocitose eritrocitária; BNP: peptídeo natriurético cerebral; TAPSE: excursão sistólica do plano do anel tricúspide; VRT: velocidade de regurgitação tricúspide; PASP: pressão sistólica da artéria pulmonar; FAC: fração de variação da área; S′: pico da velocidade sistólica do anel tricúspide; AD: átrio direito; VD: ventrículo direito.

Observou-se uma leve predominância de pacientes com RDW normal na amostra. A classe funcional e a SpO_2_ foram significativamente diferentes entre os grupos de RDW. Não houve diferença quando os pacientes foram comparados de acordo com a etiologia da HP (incluindo pacientes com ou sem doença do tecido conjuntivo, e HAP idiopática), ou com o número de medicamentos usados.

**Figura 2:**
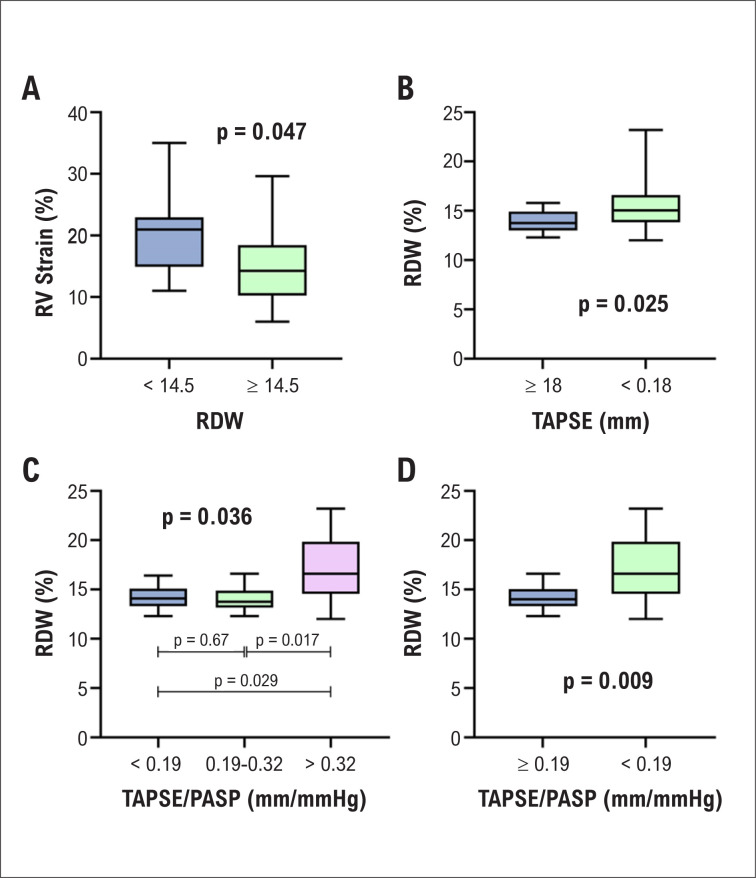
Diferença do strain do Ventrículo Direito (VD) entre grupos com Índice de Anisocitose Eritrocitária (RDW) normal e aumentada (2A). Comparação do RDW de acordo com o valor de normalidade para excursão sistólica do plano do anel tricúspide TAPSE (2B) e estratificação prognóstica pelo TAPSE/ Pressão Sistólica da Artéria Pulmonar (TAPSE/PSAP) (2C e 2D).

Entre as variáveis ecocardiográficas, somente o *strain* do VD mostrou diferença significativa (Figura 2A). O RDW foi maior nos pacientes com TAPSE alterada (<18 mm) (Figura 2B) e naqueles com derrame pericárdico (14% versus 15,4%, p=0,017).

O efeito da estratificação de risco^1^ sobre o RDW foi avaliado, considerando as variáveis ecocardiográficas TAPSE/PASP, área atrial direita, BNP e classe funcional (Tabela S2). O RDW foi diferente somente entre os grupos TAPSE/PSAP, com significância estatística entre risco baixo e alto e entre risco alto e intermediário (Figura 2C).

Quando os pacientes foram divididos em dois grupos quanto a TAPSE/PSAP (risco baixo/intermediário e alto) usando um ponto de corte de 0,19 (Figura 2D), observou-se uma diferença estatisticamente significativa no RDW.

### Sobrevida

O tempo mediano de seguimento foi 15 (10-40) meses, com um mínimo de 1 e um máximo de 43 meses. Sete pacientes foram a óbito, dos quais seis apresentavam um RDW ≥ 14,5%. Houve uma associação entre mortalidade e presença de RDW normal ou alterado (Tabela S3).

**Figura 3:**
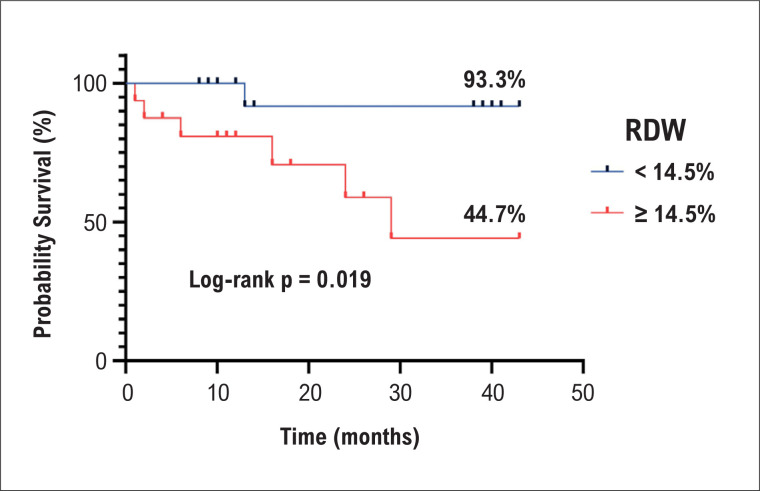
Sobrevida de Kaplan-Meier nos grupos de pacientes com Índice de Anisocitose Eritrocitária (RDW) normal e aumentado.

Os pacientes com RDW aumentado apresentaram uma sobrevida global significativamente mais curta que pacientes com RDW normal. Curvas de sobrevivência de Kaplan-Meier mostraram uma separação significativa dos dois subgrupos (Figura 3).

A análise de regressão de Cox univariada (Tabela 3) identificou o RDW ≥ 14,5% como preditor de mortalidade, bem como um *strain* do VD < 16% e uma SpO_2_ ≤ 93%. Na regressão multivariada, no entanto, nenhuma das variáveis foi preditora independente de mortalidade.

**Tabela 3:** Regressão de Cox univariada e multivariada para predizer mortalidade

Variáveis	Análise univariada		Análise multivariada	
	HR (95% CI)	p Value	HR (95% CI)	p Value
Sexo feminino	0.69 (0.07–5.79)	0.69	-	-
Idade	0.99 (0.94–1.03)	0.72	-	-
SpO_2_ ≤93%	11.37 (1.34–95.51)	0.026	8.479 (0.980–73.389)	0.052
RDW ≥14,5%	8.55 (1.02–71.66)	0.048	1.592 (0.127–19.966)	0.718
BNP >300 pg/ml	2.27 (0.35–14.55)	0.38	-	-
TAPSE <18 mm	0.98 (0.21–4.38)	0.97	-	-
VRT (m/s)	1.60 (0.892–2.869)	0.115	-	-
PASP (mmHg)	1.003 (0.97–1.037)	0.871	-	-
FAC (%)	1.002 (0.953–1.055)	0.928	-	-
S′ <9,5 cm/s	0.60 (0.113–3.190)	0.549	-	-
TAPSE/PASP <0.19 mm/mmHg	1.375 (0.262–7.220)	0.707	-	-
Área do AD >18 cm^2^	2.838 (0.861–9.351)	0.086	-	-
Strain do AD <25%	4.8 (0.56–41.7)	0.15	-	-
Strain do VD <16%	11.0 (1.3–93.3)	0.028	3.938 (0.412–37.604)	0.234
Derrame pericárdico	11.6 (2.0–65.8)	0.006	4.088 (0.551–30.322)	0.168

HR: Hazard ratio; SpO_2_: Saturação de oxigênio arterial periférico; RDW: Amplitude da Distribuição dos Glóbulos Vermelhos; BNP: Peptídeo Natriurético Cerebral; TAPSE: excursão sistólica do plano do anel tricúspide; VRT: Velocidade de Regurgitação Tricúspide; PASP: Pressão Sistólica da Artéria Pulmonar; FAC: Fração de variação da área; S′: pico da velocidade sistólica do anel tricúspide; AD: Átrio Direito; VD: Ventrículo Direito.

### Modelos para predição de mortalidade

**Figura 4:**
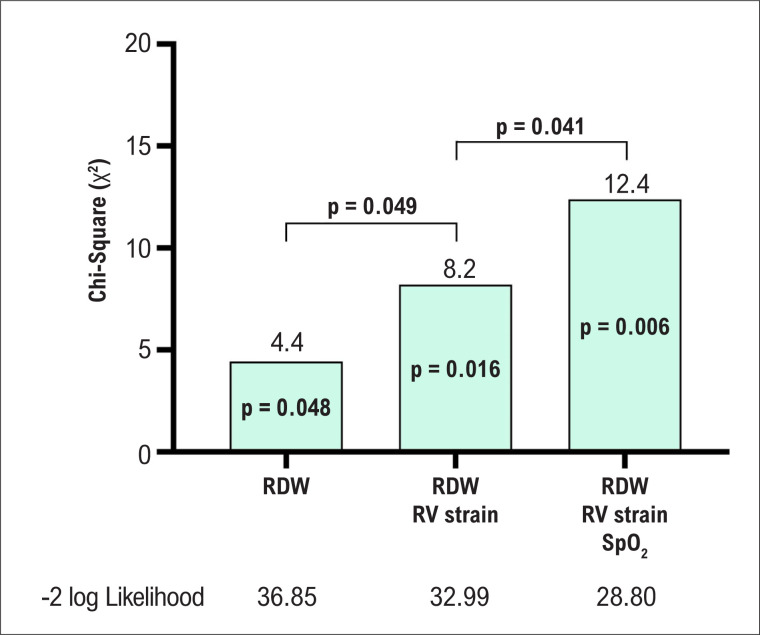
Valor incremental do strain do Ventrículo Direito (VD) e saturação periférica de oxigênio (SpO_2_) ao Índice de Anisocitose Eritrocitária (RDW) na predição de mortalidade.

O valor incremental da adição de variáveis clínicas e ecocardiográficas que foram significativas na análise univariada no modelo incluindo somente RDW também foi avaliado (Figura 4). Quando o *strain* do VD <16% foi adicionado, o modelo foi significativamente melhor em predizer mortalidade que o modelo incluindo RDW. Quando SpO_2_ ≤93% foi adicionada, o modelo foi significativamente melhor que o modelo anterior. Os p-valores de cada modelo estão apresentados na Figura 4.

**Figura 5:**
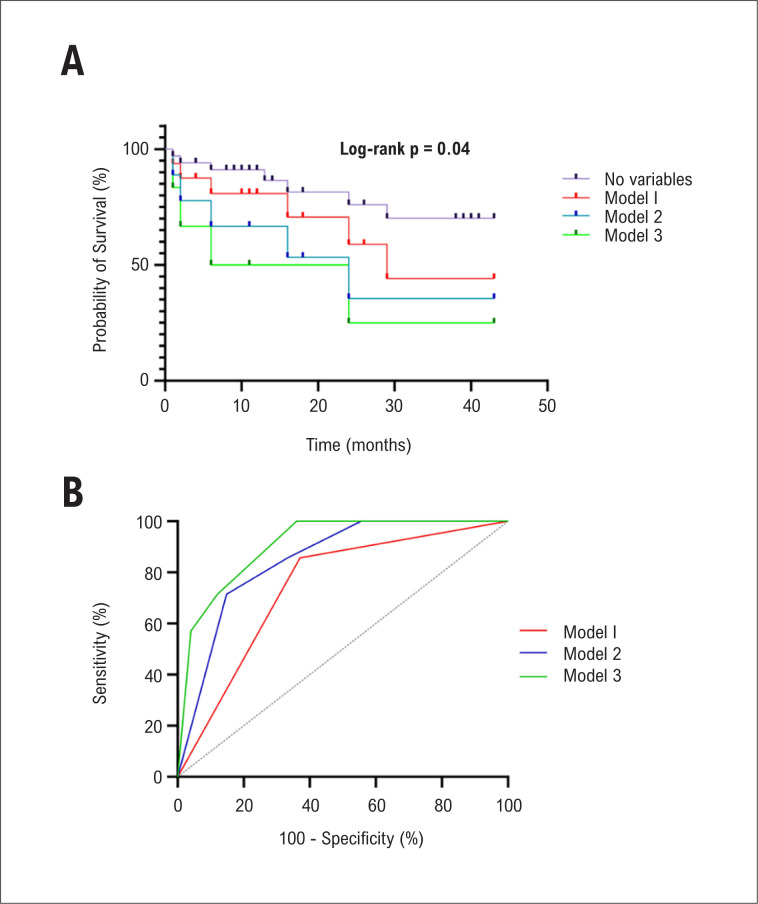
A) Comparação das curvas de sobrevida na ausência de variáveis preditoras e nos modelos 1 (RDW ≥14,5%), 2 (RDW ≥14,5% e strain do VD <16%) e 3 (RDW ≥14,5%, strain do VD <16%, e SpO_2_ ≤93%), com as respectivas probabilidades: 70,2%; 44,7%; 35,6%; e 25%; B) Curvas Características de Operação do Receptor (ROC) dos modelos 1, 2 e 3 para predição de mortalidade, com áreas sob a curva ± desvio padrão (sensibilidade, especificidade), respectivamente, de: 0,729 ± 0,087 (85,7%, 63%); 0,837 ± 0,074 (71,4%, 85,2%); e 0,909 ± 0,053 (100%, 64%); RDW: índice de anisocitose eritrocitária; VD: ventrículo direito; SpO_2_: saturação periférica de oxigênio.

A análise da curva ROC também mostrou um aumento progressivo na sensibilidade, especificidade e área sob a curva (Figura 5). De maneira semelhante, ao comparar as curvas de sobrevivência (Figura 5), observou-se uma redução significativa na probabilidade de sobrevida quando parâmetros laboratoriais, ecocardiográficos e clínicos foram avaliados.

## Discussão

Nosso estudo é o primeiro a analisar uma população composta de diferentes etiologias de HP pré-capilar, comparando anisocitose eritrocitária com marcadores ecocardiográficos avançados de lesão subclínica do VD. Foi possível avaliar não só o impacto do RDW sobre a sobrevida do paciente, como também o valor incremental de um modelo composto de parâmetros clínicos, laboratoriais e ecocardiográficos, o que é muito útil na prática clínica.

Nos pacientes estudados, o RDW não se correlacionou com hemoglobina, e não houve diferença significativa na hemoglobina entre os grupos com RDW normal e elevado. Além disso, não houve diferença no RDW quando os grupos foram separados de acordo com a etiologia da HP, mostrando que sua alteração ocorre independentemente da causa.

Estudos mostraram que o RDW é significativamente maior nos pacientes com HP secundária a diferentes etiologias, tais como DPOC, com valores de 15,1% e 13,7% nos pacientes com e sem HP, respectivamente (p<0,001);^3^ doenças do tecido conjuntivo (14,9% *versus* 13,8%, p=0,02);^8^ e tromboembolismo pulmonar (18,6% *versus* 17,0%, p=0.014).^9^

Os valores médios de quase todos os principais parâmetros ecocardiográficos relacionados ao VD também estavam alterados. O RDW mostrou uma correlação inversa com TAPSE e TAPSE/PSAP, e também se observou uma diferença significativa nos valores do RDW nos grupos com TAPSE normal e alterada. Não encontramos associação do RDW com BNP e *strain* do AE.

Um estudo com pacientes com esclerose sistêmica com ou sem HP^10^ mostrou que o RDW foi inversamente relacionado à TAPSE (ρ=-0,350; p=0,002), mas diretamente relacionado à PSAP (ρ=0,272; p=0,016) e a marcadores de sobrecarga atrial, tais como BNP (ρ=0,294; p=0,008) e *strain* global do AE (ρ=-0,396; p=0,027). Em pacientes com DPOC com e sem HP,^11^ o RDW mostrou uma correlação positiva com PSAP (r=0,594, p<0,001).

Não há, na literatura, nenhum outro estudo que correlacione o RDW na HP pré-capilar com TAPSE/PSAP, a qual é uma importante medida direta, não invasiva, do acoplamento do VD e artéria pulmonar, recentemente usada na estratificação prognóstica da HP.

Em nossa análise, observou-se uma redução significativa no *strain* do VD em pacientes com RDW aumentado (≥14,5%), o que não foi observado em outros parâmetros ecocardiográficos avaliando o VD. Assim, é possível que o *strain* do VD seja considerado um parâmetro precoce de disfunção cardíaca direita e que ele reflita a mudança no RDW.

Na literatura, há poucos estudos avaliando a relação entre RDW e o *strain* na parede livre do VD na HP. Portanto, nossos achados podem ser considerados relevantes, uma vez que esse parâmetro ecocardiográfico foi descrito como um forte preditor de desfechos de longo prazo relacionados à função sistólica em pacientes com HP.^12^

Também demonstramos que o RDW estava significativamente aumentado no grupo com derrame pericárdio, o qual, quando presente na HP, é uma variável independente associada com mortalidade.^13^ A prevalência de derrame foi similar à relatada na literatura: 25%,^14^ 15%,^15^ e 16%.^16^

Os fatores ecocardiográficos que afetam adversamente o prognóstico da HP são disfunção ventricular direita e presença de derrame pericárdico.^15^ Nesse sentido, o RDW pode estar relacionado com o prognóstico de HP, uma vez que os pacientes com um *strain* do VD pior ou com derrame apresentaram um RDW significativamente aumentado.

A razão TAPSE/PSAP foi incluída na estratificação prognóstica do risco de mortalidade de um ano (baixo, intermediário e alto) na HP.^1^ Em um estudo^17^ que estratificou os valores de TAPSE/PSAP por tercil (baixo: <0,19 mm/mmHg; intermediário: 0,19-0,32 mm/mmHg; alto: >0,32 mm/mmHg), os pacientes no tercil baixo apresentaram um status hemodinâmico, funcional e ecocardiográfico pior que pacientes nos tercis intermediário e alto.

Em nossa análise, não só o RDW foi significativamente maior no grupo com TAPSE/PSAP menor que 0,19 mm/mmHg em comparação aos pacientes acima desse valor, mas também se observou uma diferença no RDW entre os tercis baixo e alto, médio e alto, mas não entre os tercis baixo e médio. Assim, observamos que uma pior capacidade da contratilidade do VD em compensar o aumento na pós-carga foi associado a um aumento no RDW.

Essa alteração laboratorial não foi significativa quando as outras variáveis usadas para estratificação (BNP, área do AE e classe funcional) foram avaliadas. De acordo com um grupo de autores^3^ que avaliaram pacientes com DPOC e HP, o RDW se correlacionou positivamente com BNP (r=0,513, p=0,001). Esse resultado também foi demonstrado em outro estudo,^18^ em que o RDW foi maior nos pacientes com HP com BNP elevado (≥300 pg/mL) em comparação àqueles com BNP normal (<300 pg/mL) (15,0% *versus* 14,4%, p=0,0264), diferente de nossos resultados.

Por outro lado, ao comparar os grupos com RDW normal e elevado, observou-se uma diferença significativa na classe funcional, com uma predominância das classes III e II, respectivamente. Em uma coorte,^19^ valores de RDW mais altos foram observados em pacientes com classes NYHA mais altas (13,8±1,8% versus 16,5±2,9%, p<0,001). Ainda, em um estudo com 56 pacientes com hipertensão pulmonar tromboembólica crônica (HPTEC), os níveis de RDW correlacionaram-se positivamente com a classe funcional OMS (r=0,450, p=0,001).^20^

Os resultados do nosso estudo também sugeriram um valor prognóstico significativo do RDW na predição da mortalidade. Nesse sentido, uma meta-análise^21^ sugeriu que um RDW aumentado pode predizer um pior prognóstico na HP (HR=1,27, IC95% 1,11-1,45).

Em pacientes com HAP idiopática, a mortalidade por todas as causas foi significativamente pior em pacientes com RDW > 13,65% (p=0,007).^22^ Resultados similares foram encontrados em 109 pacientes com síndrome de Eisenmenger; 19,3% deles foram a óbito durante um período mediano de 4,2 anos, uma proporção similar a nossos resultados. Um RDW mais alto foi encontrado em não sobreviventes que em sobreviventes (16,9% *versus* 14,3%; p=0,015).^23^ Em um estudo prospectivo do tipo coorte^24^ de 77 pacientes com HP grupo 1 e HPTEC, o RDW médio de todas as internações foi preditivo de mortalidade (HR=1,47; IC95% 1,19-1,82).

Dada a fisiopatologia da HP, que envolve inflamação e disfunção microvascular, sua relação com o RDW é uma hipótese considerável, que foi demonstrada em nosso estudo. Além disso, a identificação de um modelo que inclui parâmetros laboratoriais, ecocardiográficos e clínicos capazes de melhor predizer a mortalidade na HP é de grande importância na prática clínica, dada a facilidade de obter esses marcadores.

O RDW é um parâmetro já incluído no hemograma, um exame solicitado rotineiramente no seguimento dos pacientes. Ainda, a SpO_2_ faz parte do exame físico dos pacientes com HP e, como confirmado em nossos resultados, é capaz de melhorar a predição de mortalidade mesmo na presença de parâmetros laboratoriais e ecocardiográficos, destacando seu elevado valor incremental e sua importância clínica como um marcador prognóstico.

A redução na saturação mediana de oxigênio em pacientes com RDW aumentado pode apoiar a hipótese de um papel da hipóxia arterial no aumento da anisocitose eritrocitária em pacientes com HP. Foi demonstrado que, nesta doença, as células na parede vascular expressam quantidades elevadas de fator induzido por hipóxia-1 alfa (HIF-1α) e fator de crescimento do endotélio vascular endotelial, que são expressos sob condições de hipóxia.^25^ Isso eleva a síntese de eritropoietina, resultando em eritrocitose.

Existem algumas limitações potenciais em nosso estudo. O pequeno tamanho amostral e o fato de a pesquisa ter sido conduzida em um único centro pode explicar a ausência de associação do RDW com algumas variáveis, tais como BNP, já descrito em outros estudos, bem como a ausência de preditores independentes de mortalidade na análise multivariada de Cox. Apesar do pequeno tamanho amostral, foi possível encontrar resultados que podem ser usados e estendidos em estudos futuros.

Outro aspecto a ser considerado é a presença de heterogeneidade etiológica na amostra. Porém, todos os participantes têm a mesma fisiopatologia em estudo, uma vez que todos eles possem HP pré-capilar.

Ainda, dadas as limitações logísticas do serviço, os pacientes não foram submetidos ao cateterismo cardíaco direito próximo aos exames laboratoriais e ecocardiográficos, o que impossibilitou a avaliação de parâmetros hemodinâmicos. O curto período de acompanhamento também deve ser considerado.

## Conclusões

Nosso estudo foi o primeiro a demonstrar uma associação da anisocitose com acoplamento ventrículo-arterial, *strain* da parede livre do VD na HP pré-capilar, bem como com presença de derrame pericárdico e sobrevida reduzida. Não existem outros estudos que avaliaram, em conjunto, RDW, *strain* do VD e SpO_2_ para predizer desfechos na HP. Esses parâmetros, de baixo custo de fácil obtenção, têm o potencial de serem usados como marcadores clínicos prognósticos nessa população de pacientes.

## *Material suplementar

Para informação adicional do Material Suplementar 1, por favor, clique aqui.

Para informação adicional do Material Suplementar 2, por favor, clique aqui.

Para informação adicional do Material Suplementar 3, por favor, clique aqui.

## References

[1] Humbert M, Kovacs G, Hoeper MM, Badagliacca R, Berger RMF, Brida M (2022). 2022 ESC/ERS Guidelines for the Diagnosis and Treatment of Pulmonary Hypertension. Eur Heart J.

[2] Simonneau G, Montani D, Celermajer DS, Denton CP, Gatzoulis MA, Krowka M (2019). Haemodynamic Definitions and Updated Clinical Classification of Pulmonary Hypertension. Eur Respir J.

[3] Yang J, Liu C, Li L, Tu X, Lu Z (2019). Red Blood Cell Distribution Width Predicts Pulmonary Hypertension Secondary to Chronic Obstructive Pulmonary Disease. Can Respir J.

[4] Zuk M, Migdal A, Dominczak J, Brzezinska-Rajszys G (2019). Usefulness of Red Cell Width Distribution (RDW) in the Assessment of Children with Pulmonary Arterial Hypertension (PAH). Pediatr Cardiol.

[5] Petrauskas LA, Saketkoo LA, Kazecki T, Saito S, Jaligam V, de Boisblanc BP (2019). Use of Red Cell Distribution Width in a Population at High Risk for Pulmonary Hypertension. Respir Med.

[6] Eroglu E, Kilicgedik A, Kahveci G, Bakal RB, Kirma C (2018). Red Cell Distribution Width and its Relationship with Global Longitudinal Strain in Patients with Heart Failure with Reduced Ejection Fraction: A Study Using Two-dimensional Speckle Tracking Echocardiography. Kardiol Pol.

[7] Mitchell C, Rahko PS, Blauwet LA, Canaday B, Finstuen JA, Foster MC (2019). Guidelines for Performing a Comprehensive Transthoracic Echocardiographic Examination in Adults: Recommendations from the American Society of Echocardiography. J Am Soc Echocardiogr.

[8] Bellan M, Giubertoni A, Piccinino C, Dimagli A, Grimoldi F, Sguazzotti M (2019). Red Cell Distribution Width and Platelet Count as Biomarkers of Pulmonary Arterial Hypertension in Patients with Connective Tissue Disorders. Dis Markers.

[9] Abul Y, Ozsu S, Korkmaz A, Bulbul Y, Orem A, Ozlu T (2014). Red Cell Distribution Width: A New Predictor for Chronic Thromboembolic Pulmonary Hypertension After Pulmonary Embolism. Chron Respir Dis.

[10] Ubertini E, Dimagli A, Giubertoni A, Zanaboni J, Bellan M, Grimoldi F (2018). Pulmonary Arterial Hypertension in Connective Tissue Disorders: Red Cell Distribution Width as a Novel Biomarker for Early Diagnosis and Follow-up. Eur Heart J.

[11] Sousa, SR, Caldeira JN, Rodrigues C, Figueiredo A, Barata FJ (2020). Red Blood Cell Distribution Width as a Potential Predictor of Pulmonary Hypertension Secondary to Chronic Obstructive Pulmonary Disease. Eur J Respir Med.

[12] Costa AA (2018). Valor Diagnóstico e Impacto Prognóstico da Deformação Miocárdica ("Strain") do Ventrículo Direito em Pacientes com Hipertensão Arterial Pulmonar e Capacidade Funcional Relativamente Preservada, Avaliados por Ecocardiografia e Ressonância Magnética Cardíaca (dissertation).

[13] Sahay S, Tonelli AR (2013). Pericardial Effusion in Pulmonary Arterial Hypertension. Pulm Circ.

[14] Benza RL, Miller DP, Gomberg-Maitland M, Frantz RP, Foreman AJ, Coffey CS (2010). Predicting Survival in Pulmonary Arterial Hypertension: Insights from the Registry to Evaluate Early and Long-Term Pulmonary Arterial Hypertension Disease Management (REVEAL). Circulation.

[15] Batal O, Khatib OF, Dweik RA, Hammel JP, McCarthy K, Minai OA (2012). Comparison of Baseline Predictors of Prognosis in Pulmonary Arterial Hypertension in Patients Surviving ≤2 Years and Those Surviving ≥5 Years After Baseline Right-sided Cardiac Catheterization. Am J Cardiol.

[16] Zhang R, Dai LZ, Xie WP, Yu ZX, Wu BX, Pan L (2011). Survival of Chinese Patients with Pulmonary Arterial Hypertension in the Modern Treatment Era. Chest.

[17] Tello K, Axmann J, Ghofrani HA, Naeije R, Narcin N, Rieth A (2018). Relevance of the TAPSE/PASP Ratio in Pulmonary Arterial Hypertension. Int J Cardiol.

[18] Todhe P, Sharma N, Ravi D, Haider S, Sravanthi MV, Ochieng P (2020). Red Cell Distribution Width Correlates with NT-Probnp in Pulmonary Hypertension. Chest.

[19] Hui M, Zhao J, Tian Z, Wang J, Qian J, Yang X (2019). Red Blood Cell Distribution Width as a Potential Predictor of Survival of Pulmonary Arterial Hypertension Associated with Primary Sjogren's Syndrome: A Retrospective Cohort Study. Clin Rheumatol.

[20] Wang W, Liu J, Yang YH, Zhai ZG, Wang C, Wang J (2016). Red Cell Distribution Width is Increased in Chronic Thromboembolic Pulmonary Hypertension. Clin Respir J.

[21] Liu J, Yang J, Xu S, Zhu Y, Xu S, Wei L (2020). Prognostic Impact of Red Blood Cell Distribution Width in Pulmonary Hypertension Patients: A Systematic Review and Meta-analysis. Medicine.

[22] Xi Q, Liu Z, Zhao Z, Luo Q (2015). Red Blood Cell Distribution Width Predicts Responsiveness of Acute Pulmonary Vasodilator Testing in Patients with Idiopathic Pulmonary Arterial Hypertension. Clin Chim Acta.

[23] Yang T, Sun YJ, Xiong CM, Zeng WJ, Ni XH, Zhao ZH (2014). Red Blood Cell Distribution Width Predicts Survival in Patients with Eisenmenger Syndrome. Clin Chem Lab Med.

[24] Smukowska-Gorynia A, Tomaszewska I, Malaczynska-Rajpold K, Marcinkowska J, Komosa A, Janus M (2018). Red Blood Cells Distribution Width as a Potential Prognostic Biomarker in Patients With Pulmonary Arterial Hypertension and Chronic Thromboembolic Pulmonary Hypertension. Heart Lung Circ.

[25] Tuder RM, Chacon M, Alger L, Wang J, Taraseviciene-Stewart L, Kasahara Y (2001). Expression of Angiogenesis-related Molecules in Plexiform Lesions in Severe Pulmonary Hypertension: Evidence for a Process of Disordered Angiogenesis. J Pathol.

