# Supplementary material for: Novo Modelo Incremental para Predição de Mortalidade na Hipertensão Pulmonar Pré-Capilar
Source: Arq Bras Cardiol. 2024 Jul 23;121(7):e20230669. [Article in Portuguese] doi: 10.36660/abc.20230669 (PMC11341211; doi:10.36660/abc.20230669)
Supplement: Translation

# Translation: New Incremental Model for Predicting Mortality in Pre-Capillary Pulmonary Hypertension

**DOI:** 10.36660/abc.20230669i

**Authors:** Andressa Alves de Carvalho, Wanessa Alves de Carvalho, Eliauria Rosa Martins, Agostinho Hermes de Medeiros, Fernando Bacal, Marcelo Dantas Tavares de Melo

**Affiliations:** 1 João Pessoa PB Brazil Universidade Federal da Paraíba, João Pessoa, PB – Brazil; 2 São Paulo SP Brazil Universidade de São Paulo Faculdade de Medicina Hospital das Clínicas Instituto do Coração, São Paulo, SP – Brazil

**Keywords:** Pulmonary Hypertension, Erythrocytes, Global Longitudinal Strain

## Abstract

**Background:**

In pulmonary hypertension (PH), the identification of easily obtainable prognostic markers associated with right ventricular (RV) dysfunction and survival is needed.

**Objective:**

To evaluate the association of red cell distribution width (RDW) with clinical, echocardiographic parameters and survival in patients with pre-capillary PH, with the development of a mortality prediction model.

**Methods:**

Observational, longitudinal, and prospective study conducted from May 2019 to December 2022. Thirty-four patients with pre-capillary PH underwent two-dimensional echocardiography and complete blood count. A cutoff point of 14.5% was considered to define RDW as altered (≥14.5%) or normal (<14.5%). P values <0.05 were considered significant.

**Results:**

The median RDW was 14.4%. There was a significant difference in peripheral arterial oxygen saturation (SpO_2_) (p=0.028), RV strain (p=0.047), and pericardial effusion (p=0.002) between the normal and elevated RDW groups. During a median follow-up of 15 months, 20.6% died. Patients with increased RDW had a shorter overall survival (44.7%, log-rank p=0.019), which was a predictor of mortality in univariate Cox regression (HR 8.55, p=0.048). The addition of RV strain <16% and SpO_2_ ≤93% to the model including RDW alone showed incremental value in predicting mortality (χ^2^=8.2, p=0.049; χ^2^=12.4, p=0.041), with increased area under the receiver operating characteristic curve (0.729 vs. 0.837 vs. 0.909) and decreased probability of survival (44.7% vs. 35.6% vs. 25%, log-rank p=0.019).

**Conclusions:**

RDW provides information on the severity of pre-capillary PH by correlating with echocardiographic parameters of RV dysfunction and mortality, which is best predicted by a model including RDW, RV strain and SpO_2_.

## Introduction

Pulmonary hypertension (PH) is defined by a mean pulmonary artery pressure ≥ 20 mmHg as assessed by right heart catheterization.^1^ Pre-capillary PH is characterized by a pulmonary wedge pressure (PWP) ≤ 15 mmHg and includes patients from groups 1, 3 and 4, some patients from group 5 and, rarely, patients from group 2 who have combined pre- and post-capillary PH.^2^

In advanced stages, PH can lead to right ventricular hypertrophy and terminal right heart failure. In this sense, there is a clear need to identify easily obtainable prognostic markers associated with right ventricular dysfunction and survival in patients with PH.

The red cell distribution width (RDW) is one of the parameters obtained from the complete blood count (CBC) and measures the variability in the volume of circulating red blood cells. When elevated, it reflects the presence of dysfunctional erythropoiesis, increased destruction, or reduced red cell lifespan.^3^ The most common cause of RDW elevation is anemia,^4^ but recent research shows that its increase is associated with several conditions, such as PH, in which it has prognostic value.^5^

There are no clear mechanisms to explain the relationship between RDW and cardiovascular disease. One of the main hypotheses is the role of chronic inflammation, which causes myelosuppression, reduces renal synthesis of erythropoietin, and triggers apoptosis of erythroid precursors in the bone marrow, increasing anisocytosis.^6^

The aim of this study was to evaluate the association of RDW with clinical, laboratory, and echocardiographic parameters in patients with pre-capillary PH, as well as its prognostic value for survival, with the development of an incremental model for mortality prediction.

## Methods

### Study design and population

This is an observational, longitudinal, and prospective study, conducted from May 2019 to December 2022. Patients with a diagnosis of pre-capillary PH confirmed by right heart catheterization, older than 18 years, and followed up at the pneumology outpatient clinic of a university hospital were included.

Exclusion criteria were: a) patients with post-capillary PH; b) presence of hemodynamic congestion on echocardiography (E/E′> 14), grade 2 or 3 diastolic dysfunction, or reduced left ventricular ejection fraction; c) left-sided structural or valvular heart disease; d) corrected or uncorrected congenital heart disease; e) inadequate echocardiographic window; f) pregnant women; g) refusal to sign the informed consent form.

The study was approved by the Research Ethics Committee of the Centro de Ciências Médicas da Universidade Federal da Paraíba, number 3,616,337, CAAE: 21291419.6.0000.8069.

### Echocardiogram

Echocardiography was performed using a GE Vivid T8 unit with a 2.5 MHz M4h-5 transducer. Images were acquired in the left lateral decubitus position according to the recommendations of the American Society of Echocardiography.^7^ Video images corresponding to three cardiac cycles were acquired. Ejection fraction was estimated using the Simpson biplane method.

Myocardial strain was assessed on a workstation using the EchoPach V204 program, and endocardial tracing was performed manually at the end of diastole. The measurement was made after the examiner checked the quality of the endocardial border tracking. If two segments were found inadequate, the examination was excluded from the study. In the apical window, sections focusing on the right ventricle (RV) and right atrium (RA) were acquired, with quantification of mean free wall strain.

### Laboratory tests

RDW was obtained from CBC, performed up to one month after two-dimensional transthoracic Doppler echocardiography. The RDW was considered altered or normal according to the RDW cut-off value (14.5%) adopted in the institution where the study was conducted. In addition, brain natriuretic peptide (BNP) levels were measured and compared with RDW.

### Outcome

Participants were included at different time points and were followed from the date of the echocardiogram until the end of the study period or the date of death. The endpoint was defined as mortality during the follow-up period.

### Bias

To reduce the risk of bias, all echocardiographic examinations were performed by a single examiner blinded to patient PH classification (group), and laboratory tests were carried out in the laboratory of the same center. Clinical variables were obtained during routine consultation by the same attending physician.

### Sample size

The sample size was defined, for convenience, by including all patients seen in the outpatient clinic of PH, who were eligible for the study.

### Statistical analysis

Continuous variables were presented as means and standard deviations (normal distribution) or medians and interquartile ranges (non-normal distribution). Categorical variables were expressed as absolute and relative frequencies. Normality of data distribution was assessed by the Kolmogorov-Smirnov test.

Parametric and non-parametric continuous variables were compared using Student's t-test for independent samples and Mann-Whitney U test, respectively. Comparisons of three or more groups of nonparametric variables were made using the Kruskal-Wallis test, with Dunn's post hoc test. The degree of correlation between two variables was determined by Spearman's correlation coefficient due to the absence of normality in the sample distribution.

Fisher's exact test was used to assess the association between RDW groups (normal/altered) and categorical variables such as mortality. Event-free survival was assessed using the Kaplan-Meier method, and curves were compared using the log-rank test.

The Cox regression analysis was used to identify the association between variables and mortality, with calculation of hazard ratio (HR) and 95% confidence interval (CI). Variables with p<0.05 in the univariate analysis were included in the multivariate model.

Sequential Cox models determined the incremental value of right ventricular (RV) strain and peripheral arterial oxygen saturation (SpO_2_) in predicting mortality by gradually adding variables to the model containing only RDW. An increment in predictive value was defined as a statistically significant increase in chi-square (χ^2^) utilizing the Omnibus Test of Model Coefficients. The −2 Log Likelihood (-2LL) was calculated to compare the capacity of the variables to predict the outcome. The improvement of the model at each stage was described by the decrease in −2LL.

Areas under the receiver operating characteristic (ROC) curve were also developed to compare the models. A p<0.05 was considered statistically significant. Statistical analyses were performed using the Statistical Package for the Social Sciences (SPSS) program, version 23. The ROC curves were evaluated with the MedCalc program. Figures were generated by GraphPad Prism 9 software.

## Results

### Patient characteristics

Thirty-four patients with pre-capillary PH were included (Table 1). During the study period, no patient was lost to follow-up. The median age of the participants was 49 years and 82.4% were female. According to the World Health Organization functional class, most patients had grade III.

**Table 1:** Clinical, laboratory, and echocardiographic parameters of the study population and the groups with normal and increased RDW

Parameters		All patients (n = 34)	Patients with RDW <14.5% (n = 18)	Patients with RDW ≥14.5% (n = 16)	p Value
Age (years)^a^		49 [37.8-66.5]	54 [43.5-68.3]	41 [37-66.5]	0.19*
Female, n (%)		28(82.4)	15(83.3)	13(81.3)	1.0†
BMI (kg/m²)^b^		26.8 ± 5.9	27.7 ± 7.2	25.7 ± 4.0	0.31††
Peripheral oxygen saturation (%)^a^		94 [92-96]	95.5 [93-97]	92.5 [90-95]	0.028*
**Functional class, n (%)**					
	I	2 (5.9)	0	2 (12.5)	0.046†
	II	8 (23.5)	7 (38.9)	1 (6.3)	
	III	24 (70.6)	11 (61.1)	13 (81.3)	
	IV	0	0	0	
**Number of medications in use, n (%)**					
	0	10(29.4)	5(27.8)	5(31.3)	0.6†
	1	10(29.4)	7(38.9)	3(18.8)	
	2	11(32.4)	5(17.8)	6(37.5)	
	3	3(8.8)	1(5.6)	2(12.5)	
**Etiology, n (%)**					
	Group I	22(64.7)	12(66.7)	10(62.5)	0.88†
	Group III	5(14.7)	3(16.7)	2(12.5)	
	Group IV	7(20.6)	3(16.7)	4(25)	
Connective tissue disease, n (%)		5(14.7)	4(22.2)	1(6.3)	0.32†
Idiopathic pulmonary arterial hypertension, n (%)		15(44.1)	6(33.3)	9(56.3)	0.41†
Hemoglobin (g/dL)^b^		13.8 ± 2.0	13.5 ± 1.3	14.3 ± 2.5	0.51††
Hematocrit (%)^a^		42.5 ± 6.3	41.7 [39.3-43.8]	43.5 [38.5-48.1]	0.4*
BNP (pg/ml)^a^		36.0 [13.2-349]	17.6 [11-109]	161.8 [21-795]	0.09*
TAPSE (m^m^)^b^		17.6 ± 5.7	19.1 ± 6.2	15.9 ± 4.7	0.11††
TRV (m/s)^b^		4.0 ± 1.0	3.9 ± 0.6	4.2 ± 1.3	0.33††
PASP (mmHg)^b^		69.4 ± 23.4	67.2 ± 23.6	71.8 ± 23.7	0.57††
FAC (%)^b^		33.1 ± 13.5	32.9 ± 14.9	33.3 ± 12.3	0.93††
S′ (cm/s)^a^		11.6 ± 3.9	11.0[10-14]	11.0[10-13]	0.42*
TAPSE/PASP (mm/mmHg)^a^		0.29 ± 0.16	0.3 [0.22-0.37]	0.19 [0.17-0.36]	0.06*
RA area (cm^2^)^a^		21.1 ± 8.1	18.3 [14.7-26.4]	19.0 [10.3-29.6]	0.81*
RA pressure (mmHg)^a^		9.1 ± 9.7	5.0[3-11.5]	8.0[3-15]	0.41*
RA strain (%)^b^		26.6 ± 17.0	27.6 ± 14.9	25.2 ± 19.8	0.72††
RV diameter (mm)^b^		41.5 ± 11.0	41.6 ± 7.2	41.5 ± 14.3	0.07††
RV strain (%)^b^		17.8 ± 6.9	20.0 ± 6.3	15.3 ± 6.8	0.047††
Pericardial effusion present, n (%)		7(20.6)	0	7(43.8)	0.002†
LVEF (%)		70.9 ± 6.6	68.8 ± 6.9	67.4 ± 5.3	0.54††
Let ventricular mass (g)		118.5 ± 37.1	131.1 ± 38.5	100.3 ± 27.4	0.02††
E/e′		6.6 ± 2.2	6.7 ± 1.7	6.3 ± 2.9	0.66††

RDW: red cell distribution width; IMC: body mass index; BNP: brain natriuretic peptide; TAPSE: tricuspid annular plane systolic excursion; TRV: tricuspid regurgitation velocity; PASP: pulmonary artery systolic pressure; FAC: fractional area change; S′: tricuspid annular peak systolic velocity; RA: right atrium; RV: right ventricle; LVEF: left ventricular ejection fraction; E/e′: ratio of early mitral inflow velocity (E) and peak early diastolic annular velocity (e’).

a Values expressed as median and interquartile range;

b Values expressed as mean and standard deviation;

* Mann-Whitney test;

† Fisher′s exact test;

†† Student′s t-test.

Regarding therapy, 61.8% were taking one or two medications. Patients who were not on optimal therapy had recently been referred for treatment. The majority were using a phosphodiesterase-5 inhibitor, followed by an endothelin receptor antagonist and/or a prostacyclin analog (Table S1).

The most common etiologies of PH were idiopathic pulmonary arterial hypertension (PAH), pulmonary thromboembolism, connective tissue disease, and chronic obstructive pulmonary disease (COPD) (Table S1).

### Laboratory and echocardiographic results

In the laboratory and echocardiographic evaluation (Table 1), the median RDW value was close to the upper normal limit. None of the patients had left heart dysfunction. Pericardial effusion was present in a minority of participants.

There was an inverse correlation of RDW with tricuspid annular plane systolic excursion (TAPSE) and TAPSE/Pulmonary artery systolic pressure (PASP) (Figure 1), but not with the other echocardiographic parameters (Table 2). RDW did not show a correlation with BNP or hemoglobin.

**Figure 1:** Correlation of RDW with TAPSE (1A) and TAPSE/PASP (1B). RDW: red cell distribution width; TAPSE: tricuspid annular plane systolic excursion; PASP: pulmonary artery systolic pressure.

**Table 2:** Spearman's correlation between RDW and echocardiographic and laboratory parameters

Laboratory and echocardiographic variables	RDW	
	ρ (correlation coefficient)	p Value
Hemoglobin	0.218	0.23
Hematocrit	0.201	0.27
BNP	0.295	0.15
TAPSE	-0.390	0.02
TRV	0.162	0.36
PASP	0.160	0.37
FAC	-0.032	0.86
S′	-0.091	0.61
TAPSE/PASP	-0.393	0.021
RA area	-0.054	0.78
RA pressure	0.167	0.35
RA strain	-0.025	0.89
RV diameter	0.001	0.99
RV strain	-0.290	0.09

RDW: red cell distribution width; BNP: brain natriuretic peptide; TAPSE: tricuspid annular plane systolic excursion; TRV: tricuspid regurgitation velocity; PASP: pulmonary artery systolic pressure; FAC: fractional area change; S′: tricuspid annular peak systolic velocity; RA: right atrium; RV: right ventricle.

There was a slight predominance of patients with normal RDW in the sample. Functional class and SpO_2_ were significantly different between the RDW groups. There was no difference when patients were compared according to PH etiology (including patients with or without connective tissue disease and idiopathic PAH) and the number of medications used.

Among the echocardiographic variables, only RV strain was significantly different (Figure 2A). RDW was higher in the group with altered TAPSE (<18 mm) (Figure 2B) and with pericardial effusion (14% versus 15.4%, p=0.017).

**Figure 2:** Difference of RV strain between groups with normal and increased RDW (2A). Comparison of RDW according to the normality value for TAPSE (2B) and prognostic stratification by TAPSE/PASP (2C and 2D). RV: right ventricular; RDW: red cell distribution width; TAPSE: tricuspid annular plane systolic excursion; PASP: pulmonary artery systolic

The effect of PH risk stratification^1^ on RDW was assessed, considering the echocardiographic variables TAPSE/PASP, right atrium (RA) area, BNP and functional class (Table S2). RDW differed only between the TAPSE/PASP groups, with significance between low and high risk and between intermediate and high risk (Figure 2C).

When patients were divided into two groups for TAPSE/PASP (low/intermediate and high risk) using a cutoff of 0.19 (Figure 2D), a statistically significant difference in RDW was found.

### Survival

The median follow-up was 15 (10-40) months, with a minimum of 1 and a maximum of 43 months. Seven patients died, six of whom had RDW ≥14.5%. There was an association between mortality and the presence of normal or altered RDW (Table S3).

Patients with increased RDW had a significantly shorter overall survival than patients with normal RDW. Kaplan-Meier overall survival curves showed a significant separation of the two subgroups (Figure 3).

**Figure 3:** Kaplan-Meier survival in the normal and increased RDW groups; RDW: red cell distribution width.

Univariate Cox regression analysis (Table 3) identified RDW ≥14.5% as a predictor of mortality, as well as RV strain <16% and SpO_2_ ≤93%. In multivariate regression, however, none of the variables was an independent predictor of mortality.

**Table 3:** Univariate and multivariate Cox regression for predicting mortality

Variables	Univariate Analysis		Multivariate Analysis	
	HR (95% CI)	p Value	HR (95% CI)	p Value
Female	0.69 (0.07–5.79)	0.69	-	-
Age	0.99 (0.94–1.03)	0.72	-	-
SpO_2_ ≤93%	11.37 (1.34–95.51)	0.026	8.479 (0.980–73.389)	0.052
RDW ≥14,5%	8.55 (1.02–71.66)	0.048	1.592 (0.127–19.966)	0.718
BNP >300 pg/ml	2.27 (0.35–14.55)	0.38	-	-
TAPSE <18 mm	0.98 (0.21–4.38)	0.97	-	-
TRV (m/s)	1.60 (0.892–2.869)	0.115	-	-
PASP (mmHg)	1.003 (0.97–1.037)	0.871	-	-
FAC (%)	1.002 (0.953–1.055)	0.928	-	-
S′ <9.5 cm/s	0.60 (0.113–3.190)	0.549	-	-
TAPSE/PASP <0.19 mm/mmHg	1.375 (0.262–7.220)	0.707	-	-
RA area >18 cm^2^	2.838 (0.861–9.351)	0.086	-	-
RA strain <25%	4.8 (0.56–41.7)	0.15	-	-
RV strain <16%	11.0 (1.3–93.3)	0.028	3.938 (0.412–37.604)	0.234
Pericardial effusion	11.6 (2.0–65.8)	0.006	4.088 (0.551–30.322)	0.168

HR: hazard ratio; SpO_2_: peripheral arterial oxygen saturation; RDW: red cell distribution width; BNP: brain natriuretic peptide; TAPSE: tricuspid annular plane systolic excursion; TRV: tricuspid regurgitation velocity; PASP: pulmonary artery systolic pressure; FAC: fractional area change; S′: tricuspid annular peak systolic velocity; RA: right atrium; RV: right ventricle.

### Models for mortality prediction

The incremental value of adding clinical and echocardiographic variables that were significant in the univariate analysis to the model including RDW only was also evaluated (Figure 4). When RV strain <16% was added, the model was significantly better at predicting mortality than the model including RDW. When SpO_2_ ≤93% was added, the model was significantly better than the previous model. The p-values for each model are shown in Figure 4.

**Figure 4:** Incremental value of RV strain and SpO_2_ to RDW in predicting mortality. RV: right ventricle; SpO_2_: peripheral arterial oxygen saturation; RDW: red cell distribution width.

ROC curve analysis also showed a progressive increase in sensitivity, specificity, and area under the curve (Figure 5). Similarly, when comparing the survival curves (Figure 5), there was a significant reduction in the probability of survival when laboratory, echocardiographic and clinical parameters were evaluated.

**Figure 5:** Comparison of survival curves in the absence of predictor variables and in models 1 (RDW ≥14.5%), 2 (RDW ≥14.5% and RV strain <16%), and 3 (RDW ≥14.5%, RV strain <16%, and SpO_2_ ≤93%), with probabilities, respectively, of: 70.2%, 44.7%, 35.6%, and 25%. B) Receiver operator characteristic curves of models 1, 2 and 3 for mortality prediction, with areas under the curve ± SD (sensitivity, specificity), respectively, of: 0.729 ± 0.087 (85.7%, 63%); 0.837 ± 0.074 (71.4%, 85.2%); and 0.909 ± 0.053 (100%, 64%). RDW: Red Cell Distribution Width; RV: Right Ventricle; SpO_2_: Peripheral Arterial Oxygen Saturation.

## Discussion

Our study is the first to analyze a population composed of different etiologies of pre-capillary PH, comparing erythrocyte anisocytosis with advanced echocardiographic markers of subclinical RV injury. It was possible to evaluate not only the impact of RDW on patient survival, but also the incremental value of a model composed of clinical, laboratory and echocardiographic parameters, which is very useful in clinical practice.

In the patients studied, RDW did not correlate with hemoglobin, and there was no significant difference in hemoglobin between the groups with normal and elevated RDW. Thus, it can be excluded that anisocytosis was influenced by hemoglobin levels in our results. Moreover, there was no difference in RDW when the groups were separated according to the etiology of PH, showing that its change occurs regardless of the cause.

Studies have shown that RDW is significantly higher in patients with PH secondary to different etiologies, such as COPD, with values of 15.1% and 13.7% in patients with and without PH, respectively (p<0.001);^3^ connective tissue diseases (14.9% *versus* 13.8%, p=0.02);^8^ and pulmonary thromboembolism (18.6% *versus* 17.0%, p=0.014).^9^

Mean values of almost all key echocardiographic parameters related to the right ventricle were altered. RDW showed an inverse correlation with TAPSE and TAPSE/PASP, and there was also a significant difference in the values of RDW in the normal and altered TAPSE groups. We found no association of RDW with BNP and RA strain.

A study with patients with systemic sclerosis with or without PH^10^ showed that RDW was inversely related to TAPSE (ρ=-0.350; p=0.002), but directly related to PASP (ρ=0.272; p=0.016) and to markers of atrial overload such as BNP (ρ=0.294; p=0.008) and RA global strain (ρ=-0.396; p=0.027). In COPD patients with and without PH,^11^ RDW showed a positive correlation with PASP (r=0.594, p<0.001).

There is no other study in the literature correlating RDW in pre-capillary PH with TAPSE/PASP, which is an important direct noninvasive measure of RV-arterial coupling recently used in prognostic stratification of PH.

In our analysis, RV strain was significantly reduced in patients with increased RDW (≥14.5%), which was not observed in the other echocardiographic parameters assessing the RV. Thus, there is a possibility that RV strain is considered an early parameter of right heart dysfunction and that it reflects the change in RDW.

In the literature, there is a paucity of studies evaluating the relationship between RDW and RV free wall strain in PH. Therefore, our findings can be considered relevant since this echocardiographic parameter has been described as a strong independent predictor of long-term outcomes related to systolic function in patients with PH.^12^

We also demonstrated that RDW was significantly increased in the group with pericardial effusion, which when present in PH is a variable independently associated with mortality.^13^ The prevalence of effusion was similar to that reported in the literature: 25%,^14^ 15%^15^ and 16%.^16^

The echocardiographic factors that adversely affect the prognosis of PH are right ventricular dysfunction and the presence of pericardial effusion.^15^ In this sense, RDW may be related to prognosis in PH, as patients with worse RV strain or with effusion had significantly increased RDW.

The TAPSE/PASP ratio has been included in the prognostic stratification of 1-year mortality risk (low, intermediate, and high) in PH.^1^ In a study^17^ that stratified TAPSE/PASP values by tertile (low: <0.19 mm/mmHg; intermediate: 0.19-0.32 mm/mmHg; high: >0.32 mm/mmHg), patients in the low tertile had significantly worse hemodynamic, functional, and echocardiographic status than patients in the intermediate and high tertiles.

In our analysis, not only was RDW significantly higher in the group with TAPSE/PASP less than 0.19 mm/mmHg compared with patients above this value, but there was also a difference in RDW between the low and high, middle and high tertiles, but not between the low and middle tertiles. Thus, we found that a worse ability of RV contractility to compensate for the increase in afterload was associated with an increase in RDW.

This laboratory change was not significant when the other variables used for stratification (BNP, RA area and functional class) were evaluated. According to a group of authors^3^ who evaluated patients with COPD and PH, RDW correlated positively with BNP (r=0.513, p=0.001). This result was also demonstrated by another study^18^ in which the RDW was higher in patients with PH with elevated BNP (≥300 pg/mL) compared to those with normal BNP (<300 pg/mL) (15.03% *versus* 14.36%, p=0.0264), which is different from our results.

On the other hand, when comparing the groups with elevated and normal RDW, there was a significant difference in functional class, with a predominance of classes III and II, respectively. In a cohort,^19^ higher RDW values were found in patients with higher NYHA classes (13.8±1.8% versus 16.5±2.9%, p<0.001). Similarly, in a study of 56 patients with chronic thromboembolic pulmonary hypertension (CTEPH), RDW levels were found to be positively correlated with WHO functional class (r=0.450, p=0.001).^20^

The results of our research also suggest a significant prognostic value of RDW in predicting mortality. In this regard, a meta-analysis^21^ suggested that increased RDW may predict a worse prognosis in PH (HR=1.27, 95% CI 1.11-1.45).

In patients with idiopathic PAH, all-cause mortality was significantly worse in patients with RDW >13.65% (p=0.007).^22^ Similar results were found in 109 patients with Eisenmenger's syndrome, 19.3% of whom died during a median follow-up of 4.2 years, a proportion similar to our results. A higher RDW was found in non-survivors than in survivors (16.9% *versus* 14.3%, p=0.015).^23^ In a prospective cohort study^24^ of 77 patients with group 1 PH and CTEPH, the mean RDW of all hospitalizations was predictive of mortality (HR=1.47; 95% CI 1.19-1.82).

Given the pathophysiology of PH, which involves inflammation and microvascular dysfunction, its relationship with RDW is a considerable hypothesis, which was demonstrated in our study. Moreover, the identification of a model that includes laboratory, echocardiographic and clinical parameters capable of better predicting mortality in PH is fundamental in medical practice, given the ease of obtaining these markers.

RDW is a parameter already included in the CBC, a test routinely requested in the follow-up of patients. In addition, peripheral oxygen saturation is part of the physical examination of patients with PH and, as confirmed in our results, is able to improve the prediction of mortality even in the presence of laboratory and echocardiographic parameters, highlighting its high incremental value and clinical importance as a prognostic marker.

The reduced median oxygen saturation in patients with increased RDW may support the hypothesis of the role of arterial hypoxia in increasing erythrocyte anisocytosis in patients with PH. It has been shown that in this disease, cells in the vascular wall overexpress hypoxia-inducible factor 1-alpha (HIF-1α) and vascular endothelial growth factor, which are expressed under hypoxic conditions.^25^ This elevates erythropoietin synthesis, resulting in erythrocytosis.

There are also some potential limitations of our study. The small sample size and the fact that the research was conducted in a single center may explain the lack of association of RDW with some variables, such as BNP, already described in other studies, as well as the absence of independent predictors of mortality in the multivariate Cox analysis. Despite the small sample size, it was possible to find results that can be used and extended in future studies. Another aspect to consider is the presence of etiologic heterogeneity in the sample, but all participants have the same pathophysiology under study, as they all have pre-capillary PH. In addition, due to logistical limitations of the center, patients did not undergo right heart catheterization close to the laboratory and echocardiographic exams, which prevented the evaluation of hemodynamic parameters. The short follow-up period is also an aspect to be considered.

## Conclusions

Our study was the first to demonstrate that there is an association of anisocytosis with ventricular-arterial coupling, RV-free wall strain in pre-capillary PH, and also with the presence of pericardial effusion and reduced survival. There are no other studies that evaluated RDW, RV strain and SpO_2_ together to predict outcome in PH. These parameters, which are inexpensive and easy to obtain, have the potential to be used as clinical prognostic markers in this patient population.

## *Supplemental Materials

For additional information Supplemental Material 1, please click here.

For additional information Supplemental Material 2, please click here.

For additional information Supplemental Material 3, please click here.
